# Expansion of CD16-Negative Natural Killer Cells in the Peripheral Blood of Patients with Metastatic Melanoma

**DOI:** 10.1155/2011/316314

**Published:** 2011-02-23

**Authors:** Shernan G. Holtan, Douglas J. Creedon, Michael A. Thompson, Wendy K. Nevala, Svetomir N. Markovic

**Affiliations:** ^1^Division of Hematology, Department of Medicine, Mayo Clinic Graduate School of Medicine, 200 First Street SW, Rochester, MN 55905, USA; ^2^Department of Oncology, Mayo Clinic Graduate School of Medicine, 200 First Street SW, Rochester, MN 55905, USA; ^3^Department of Obstetrics and Gynecology, Mayo Clinic Graduate School of Medicine, 200 First Street SW, Rochester, MN 55905, USA; ^4^Department of Immunology, Mayo Clinic Graduate School of Medicine, 200 First Street SW, Rochester, MN 55905, USA

## Abstract

Altered natural killer (NK) cell function is a component of the global immune dysregulation that occurs in advanced malignancies. Another condition associated with altered NK homeostasis is normal pregnancy, where robust infiltration with CD16− CD9+ NK cells can be identified in decidual tissues, along with a concomitant expansion of CD16− NK cells in the maternal peripheral blood. In metastatic melanoma, we identified a similar expansion of peripheral blood CD16− NK cells (median 7.4% in 41 patients with melanoma compared with 3.0% in 29 controls, *P* < .001). A subset of NK cells in melanoma patients also expresses CD9, which is characteristically expressed only on NK cells within the female reproductive tract. Expansion of CD16− NK cells was associated with elevated plasma transforming growth factor-beta (TGF-*β* levels (median 20 ng/ml, Spearman's *ρ* = 0.81, *P* = .015)). These findings suggest the possibility of exploring anti-TGF-*β* therapy to restore NK function in melanoma.

## 1. Introduction

Natural killer (NK) cells are a critical component of innate immunity and tumor immunosurveillance in melanoma and other malignancies [1]. Impairment of NK cytolytic function has previously been described in melanoma [2, 3]. Another physiologic condition associated with a shift in NK homeostasis toward a noncytolytic phenotype is normal human pregnancy, where expansion of weakly cytotoxic CD56bright CD16dim/− NK cells (hereafter referred to as CD16− NK cells to distinguish from CD56bright NK cells, which may represent predecessors to CD16+ mature NK cells [4]) can be seen both in the peripheral blood [5] of pregnant women and enriched at the fetomaternal interface [6]. Specialized decidual NK cells at the fetomaternal interface serve an immunoregulatory/angiogenic function to support placentation and are phenotypically identified by the expression of CD9, a tetraspanin involved in cell adhesion [7]. Mature peripheral blood CD16+ NK cells can be transformed into CD16− CD9+ NK cells with a decidual phenotype after prolonged exposure to transforming growth factor-beta (TGF-*β* [8], a cytokine with pleiotropic immunologic effects including potential regulation of NK function at the host/tumor interface [9].) Here, we test the hypothesis that CD16− NK cells are expanded in the peripheral blood of melanoma patients as previously described in normal pregnancy. Additionally, we assessed CD9 expression on peripheral blood NK cells and determined the presence of an association with elevated TGF-*β* levels in patients with metastatic melanoma.

## 2. Materials and Methods

### 2.1. Collection of Patient Biospecimens

NK subset analysis was performed on the peripheral blood of 41 patients with untreated metastatic melanoma, 39 of whom had plasma available for multiplex cytokine arrays. For comparison, NK subsets were also analyzed in 29 healthy control individuals. Samples from patients with metastatic melanoma (newly diagnosed, previously untreated) as well as healthy volunteers/controls were collected under separate specimen banking protocols. Both protocols were reviewed and approved by the Mayo Clinic Institutional Review Board for use in these studies. Written informed consent was obtained from participants for banking of blood and tissue samples, and Mayo Clinic IRB approvals were granted for the specific studies detailed in this work. Peripheral venous blood (50–90 mL) was drawn into heparinized Vacutainer tubes that were processed and separated into plasma and peripheral blood mononuclear cells (PBMCs) following gradient centrifugation using Ficoll-Paque (GE Healthcare, Uppsala, Sweden). Plasma was collected and immediately frozen at -80°C in 1 mL aliquots. The PBMCs were collected, washed in phosphate buffered saline, counted, diluted to 1 × 10^7^/mL, and viably frozen in 90% cosmic calf serum (Hyclone Inc., Logan, UT) and 10% dimethylsulfoxide (Sigma, St. Louis, MO).

### 2.2. Evaluation of NK Cell Subsets by Flow Cytometry

Previously frozen PBMCs (0.5–1.0 × 10^6^ cells/mL) from patients with metastatic melanoma as well as healthy control patients were used for determination of NK cell subsets. Four-color flow cytometry was performed using an LSRII flow cytometer (Becton Dickinson), and CellQuest software (Becton Dickinson, San Jose, CA) was used for data analysis. The following anti-human monoclonal antibodies were used in PBMC immunophenotyping for flow cytometry: anti-CD3-PE-Cy5, anti-CD56-phycoerythrin, anti-CD16-Alexa Fluor 647, and anti-CD9 fluorescein isothiocyanate (BD Pharmingen).

### 2.3. Plasma Cytokine, Chemokine, and Growth Factor Concentrations

Protein levels for cytokines, chemokines, and growth factors, including IL-1*β*, IL-1ra, IL-2, IL-4, IL-5, IL-6, IL-7, IL-8, IL-10, IL-12p70, IL-13, IL-15, IL-17, G-CSF, GM-CSF, IFN-*γ*, IP-10, MCP-1, MIP-1*α*, MIP-1*β*, TNF-*α*, and VEGF, were measured using either the Bio-Plex cytokine assay (Bio-Rad, Hercules, CA, 28 patients) or Invitrogen BioSource multiplex cytokine assay (Invitrogen Corporation, Carlsbad, CA, 11 patients) as per manufacturer's instructions. Briefly, patient plasma was diluted 1 : 4 in dilution buffer and 50 *μ*L was added to washed, fluorescently dyed microspheres (beads) to which biomolecules of interest are bound. The beads and diluted patient plasma were incubated for 30 minutes at room temperature with agitation. After the incubation, the beads were washed in wash buffer and placed in 25 *μ*L of detection antibody and incubated for 30 minutes as described above. After washing, the beads were placed in streptavidin-phycoerythrin, incubated, and washed a final time. The bound beads were resuspended in 125 microliters of assay buffer and read with the Luminex plate reader (Bio-Rad, Hercules, CA). Protein concentrations were determined using a standard curve generated using the high PMT concentrations with sensitivity from 10–1000 pg/mL. 

### 2.4. Determination of Plasma TGF-*β* Levels by ELISA

Plasma levels of TGF-*β*1 were determined by ELISA using a duoset antibody pair (R&D Systems, Minneapolis, MN) as per manufacturer's instructions. 

### 2.5. Statistical Analysis

Differences in percentage of peripheral blood CD16− NK cells between healthy subjects and patients with metastatic melanoma were determined by the Mann–Whitney U test. Correlations between TGF-*β* levels and the percentage of NK cell subsets ware determined by Spearman's *ρ*. Correlations between cytokines were estimated by the pairwise method.

## 3. Results and Discussion

**Figure 1:**
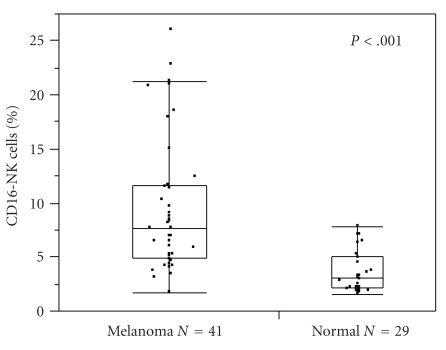
Patients with metastatic melanoma have expanded peripheral blood CD16− NK cell pools. Box-plot with quartiles depicting percentage of CD16− NK cells in peripheral blood from 41 metastatic melanoma patients (median 7.6%) compared with 29 healthy controls (median 3.1%, Mann–Whitney *P* < .001).

**Figure 2:**
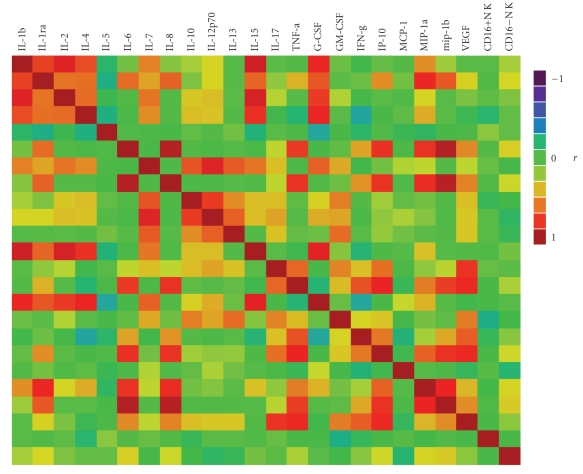
Color map on pairwise correlations between NK subsets and plasma cytokines and growth factors. None of the tested multiplex cytokines or growth factors were strongly associated with the percentages of CD16+ or CD16− NK cells.

Circulating CD16− NK cells were expanded 2.5-fold in patients with melanoma compared with healthy controls (Figure 1, median 7.4% compared with 3.0%, *P* < .001). The Median CD16+ NK cell count was 0.56 × 10^9^ per liter (range 0.03–1.53 × 10^9^ per liter), and the median CD16− NK cell count was 0.11 × 10^9^ per liter (range 0.01–0.50 × 10^9^ per liter) in patients with metastatic melanoma, within range of published values for NK cells in healthy adults [10]. None of the tested multiplex plasma cytokines or growth factors correlated strongly with the distribution of NK cells subsets (Figure 2). Median and range of these parameters are listed in Table 1. 

**Figure 3:**
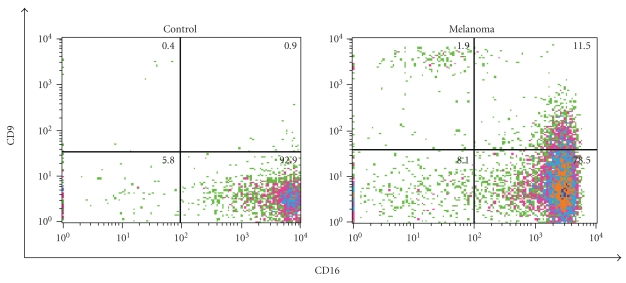
CD9 expression on NK cells in patients with melanoma. Scatterplot demonstrating CD9 expression in a subset of peripheral blood CD16− NK cells from patients with metastatic melanoma (right). CD9, a marker specific for decidual NK cells is, absent on NK cells from healthy individuals (left). Gating is set on CD3− CD56+ lymphocytes. Numbers represent percentages of total NK cells in each quadrant.

**Figure 4:**
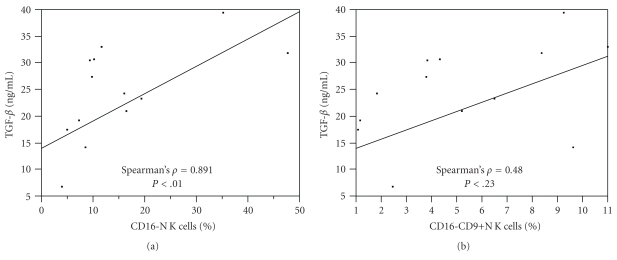
Correlation of TGF-*β* with (a) the CD16− NK cell subset and (b) the CD16− CD9+ subset. Expansion of the CD16− phenotype NK cell subset was strongly correlated with plasma levels of TGF-*β* in these patients. A moderate correlation between CD16− CD9+ NK cells and TGF-*β* did not meet statistical significance.

Normal peripheral blood CD16+ NK cells incubated in culture with TGF-*β* result in a transition to a CD16− CD9+ phenotype resembling decidual NK cells [8]. Since, like pregnancy [11], metastatic melanoma is a condition associated with elevated TGF-*β* levels [12], we next sought to evaluate whether NK cells in the peripheral blood of patients with metastatic melanoma might also express CD9 or exhibit any subset correlation with plasma levels of TGF-*β*. Eight untreated melanoma patients had 14 unique samples of PBMCs available for further phenotypic NK testing with CD9 and corresponding plasma for TGF-*β* determination. Representative plots from the experiments demonstrating CD16− CD9+ NK cells in the peripheral blood of metastatic melanoma patients are shown in Figure 3. CD9 was expressed on a median of 24% of peripheral blood NK cells in patients with metastatic melanoma (Figure 3, range 13.4–37.9%). This alteration in NK homeostasis toward a CD16− phenotype was strongly correlated with plasma levels of TGF-*β* in these patients (Figure 4(a), median 20 ng/ml, Spearman's *ρ* = 0.81, *P* = .015). A moderate correlation between CD16− CD9+ NK cells and TGF-*β* was observed that did not meet statistical significance (Figure 4(b), Spearman's *ρ* = 0.48, *P* = .23).

Bias of NK subsets toward a less cytotoxic and more immunoregulatory phenotype may be a reflection of an overall immunosuppressive milieu in patients with advanced malignancies. The association of expanded CD16− NK cells with elevated TGF-*β* levels has also been identified in patients with esophageal squamous cell carcinoma [13] and gastric cancer [14]. Other examples of altered NK homeostasis in cancer exist, including relative expansion of weakly cytolytic CD16− NK cells in patients with hepatocellular carcinoma compared with healthy controls [15], in the peripheral blood and peritoneal fluid of ovarian cancer patients [16], and in the stroma of nonsmall-cell lung cancer primary tumors [17]. Our results suggest that CD16− CD9+ NK cells may not be specific for the female reproductive tract as once thought, but may instead be reflective of systemic immunomodulation that occurs in melanoma and potentially other conditions associated with high levels of TGF-*β*. Our findings will require validation but preliminarily support further investigation into anti-TGF-*β* therapy as a part of cancer immunotherapy. 

Recent reports have begun to elucidate the mechanisms by which TGF-*β* suppresses NK function. The NK cell activating receptor NKG2D has recently been described as downregulated by TGF-*β* in cancer patients [18]. Additionally, in the presence of TGF-*β* NK, cells stimulated through CD16 show reduced *T-BET* expression and diminished production of interferon-gamma and tumor necrosis factor-alpha, further demonstrating the suppressive effects of TGF-*β* on NK cells. In melanoma, we have shown that chemotherapeutic intervention with bevacizumab, carboplatin, and paclitaxel can at least transiently alter an individuals' immunologic response to the tumor [19]. However, most patients with metastatic melanoma eventually succumb to complications of the cancer that involve systemic dysregulation of immunity, which appears to be manifest in part by altered NK cell homeostasis. In future studies, we will determine whether the observed alteration in NK homeostasis is prognostic or predictive of treatment response and survival in metastatic melanoma. 

## 4. Conclusion

Our results suggest that identification and modulation of an immunosuppressive cytokine milieu, for example, high plasma levels of TGF-*β* might allow for restoration of endogenous antitumor immunity, including recovery of NK cytolytic function, and ultimately improve outcomes of melanoma therapy.

## Figures and Tables

**Table 1:** Absolute NK cells counts and plasma cytokine/growth factor levels in patients with metastatic melanoma. Cell counts are measured in cells ×10^9^ per liter, and plasma cytokines/growth factors are measured in picograms/milliliter.

	Median	Minimum	Maximum
Absolute NK cell count	0.64	0.13	1.66
Absolute CD16+ NK	0.56	0.03	1.53
Absolute CD16− NK	0.11	0.01	0.50
IL-1b	3.22	0.00	1216.46
IL-1ra	80.90	0.19	4130.51
IL-2	7.42	0.00	463.38
IL-4	3.95	0.00	129.84
IL-5	1.02	0.00	17.22
IL-6	11.83	0.41	2307.25
IL-7	5.38	0.58	250.24
IL-8	11.45	0.06	2439.86
IL-10	4.22	0.00	175.74
IL-12p70	6.88	0.00	1637.86
IL-13	6.07	0.00	162.68
IL-15	9.22	0.00	709.06
IL-17	6.36	0.00	96.60
TNF-*α*	13.28	0.00	439.51
G-CSF	16.22	0.42	622.01
GM-CSF	70.58	0.00	419.91
IFN-*γ*	8.85	0.00	325.81
IP-10	77.56	0.73	3679.56
MCP-1	46.28	0.02	19679.10
MIP-1a	8.11	0.00	907.85
MIP-1b	79.34	0.27	13000.21
VEGF	15.32	0.00	161.15
